# Persistent Hypoglossal Artery

**DOI:** 10.5334/jbsr.3644

**Published:** 2024-08-28

**Authors:** Lucas Dekesel, Thiebault Saveyn, Benjamin Leenknegt

**Affiliations:** 1 AZ Sint-Lucas, Ghent, Belgium; 2 AZ Sint-Lucas, Ghent, Belgium; 3 AZ Sint-Lucas, Ghent, Belgium

**Keywords:** Carotid-vertebral anastomoses, persistent hypoglossal artery, congenital variant

## Abstract

*Teaching point:* Persistent hypoglossal artery is an extremely rare anatomical variant but has diagnostic and therapeutic relevance.

## Case History

**Figure 1:**
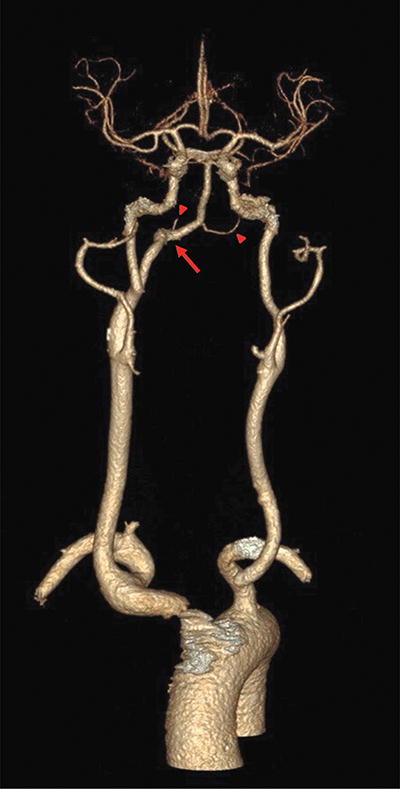
3D reconstruction of CT-angiography showing a right persistent hypoglossal artery (arrow) and hypoplastic vertebral arteries (arrowheads).

**Figure 2:**
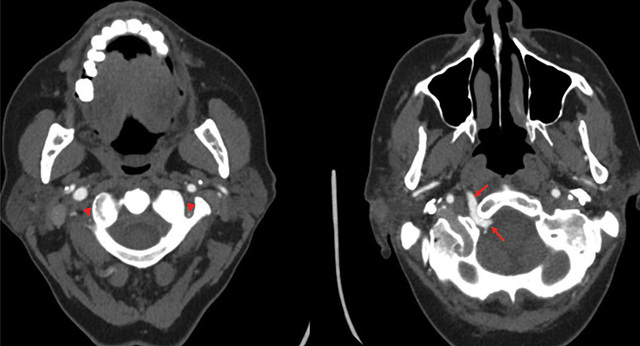
Axial CT-angiogram images demonstrating hypoplastic vertebral arteries (arrowheads) and a right persistent hypoglossal artery (arrows) coursing through a widened hypoglossal canal.

A left frontal punctiform ischemic cerebral infarct was incidentally detected on an MRI for the evaluation of brain metastases in a 64-year-old female patient with known breast cancer. She experienced no neurological complaints. Further cerebrovascular workup included a CT-angiography to rule out vascular stenoses. The CT-angiogram showed a hypoglossal artery arising from the cervical segment of the right internal carotid artery in front of the C1–C2 vertebral space and passing through the hypoglossal canal to merge with the basilar artery (Figures 1, 2). Both vertebral arteries were largely hypoplastic. The circle of Willis and internal carotid arteries were patent.

## Discussion

A persistent hypoglossal artery (PHA) is the second-most-common type of a persistent carotid-vertebrobasilar anastomosis (PCVA), with a reported prevalence of 0.02–0.09%. It is bilateral in 1.4% of cases and slightly more frequent in women [1]. PCVAs are variant anatomical communications between the anterior and the posterior circulation due to failure of these communicating vessels to regress during embryogenesis. These anastomoses are named based on their adjacent structures, and they include the most frequent persistent trigeminal artery (0.1–0.6%), PHA, proatlantal intersegmental artery, persistent dorsal ophthalmic artery, persistent olfactory artery, and the controversial persistent otic artery.

The diagnosis of a PHA is made when an anomalous artery originates from the internal carotid artery at the level of C1–C3 vertebral bodies and courses through the hypoglossal canal and anastomoses with the basilar artery. Associated widening of the hypoglossal canal can be observed. Although variable, vertebral arteries and posterior communicating arteries are usually hypoplastic, making the PHA the main feeder for the posterior circulation. This has major repercussions both therapeutically and diagnostically: Temporarily clamping the ipsilateral carotid artery (e.g. during endarterectomy) could cause serious cerebral ischemic risk, and emboli originating from the anterior circulation could cause both anterior and posterior strokes in the presence of a PHA. Given the left-sided frontal cerebral infarct in our case, it is unlikely that this infarct is related to the contralateral PHA. Although most often detected incidentally, PCVAs may rarely become symptomatic. Aneurysm formation, glossopharyngeal neuralgia, and hypoglossal nerve palsy can be caused by PHAs, probably due to hemodynamic changes and nerve irritation. Overall, PHA is considered to be the PCVA that is most often associated with pathology, making it an important structure to diagnose and report, both in acute settings and in patients with atypical neurological symptoms.
